# Cancer Stem Cells are Actually Stem Cells with Disordered Differentiation: the Monophyletic Origin of Cancer

**DOI:** 10.1007/s12015-023-10508-2

**Published:** 2023-01-17

**Authors:** Qiankun Luo, Pan Liu, Pengfei Yu, Tao Qin

**Affiliations:** Department of Hepatobilliary and Pancreatic Surgery, Zhengzhou University People’s Hospital, Henan Provincial People’s Hospital, Henan University People’s Hospital, Jinshui District, No. 7, Weiwu Rd., Zhengzhou, 450003 Henan China

**Keywords:** Cancer stem cells, Inflammatory response, Tumor origin, Differentiation, Tissue repair signal

## Abstract

Cancer stem cells (CSCs) play an important role in cancer development. Based on advancements in CSC research, we propose a monophyletic model of cancer. This model is based on the idea that CSCs are stem cells with disordered differentiation whose original purpose was to repair damaged tissues. Inflammatory responses and damage repair signals are crucial for the creation and maintenance of CSCs. Normal quiescent stem cells are activated by environmental stimulation, such as an inflammatory response, and undergo cell division and differentiation. In the initial stage of cancer development, stem cell differentiation leads to heteromorphism due to the accumulation of gene mutations, resulting in the development of metaplasia or precancerosis. In the second stage, accumulated mutations induce poor differentiation and lead to cancer development. The monophyletic model illustrates the evolution, biological behavior, and hallmarks of CSCs, proposes a concise understanding of the origin of cancer, and may encourage a novel therapeutic approach.

**Graphical Abstract:**

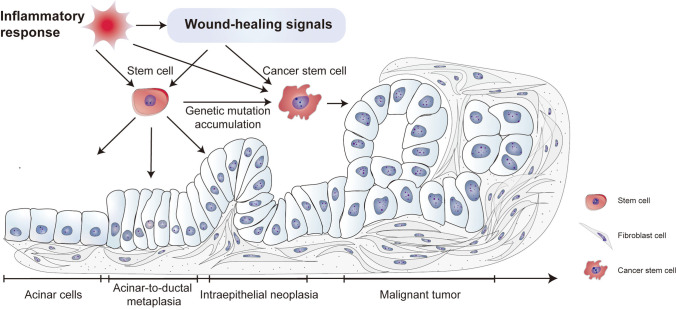

## Introduction

According to the 2022 cancer statistics, cancer is the second leading cause of death after heart disease, comprising 21% of all deaths [1]. Even though genomics, transcriptomics, epigenomics, proteomics, and metabolomics have been employed to identify diverse hallmarks of cancer, targeted therapeutic strategies for cancer have achieved little efficacy [2, 3]. Understanding cancer remains a substantial challenge. For a long time, cancer was thought to be a “wound” that never heals, since many mesenchymal stem cells (MSCs) are recruited to the tumor microenvironment in a manner similar to the repair behaviors observed in damaged tissues [4, 5]. Current studies suggest that cancers arise from normal cells that accumulate oncogenic mutations. As only stem cells exhibit a lifelong capability for division and self-renewal, cancer stem cells (CSCs), which develop from stem cells, are considered the origin of cancer. CSCs exhibit striking similarities to normal stem cells in terms of differentiation, long-term proliferation (self-renewal), drug resistance, and anti-apoptosis [6, 7]. CSCs have been identified in tumors of the liver, pancreas, breast, brain, lung, and ovary. On the other hand, the classical CSCs model has faced a series of confusions and controversies regarding CSCs’ heterogenous origin, cell proportion, uncertain cell markers, and the genomic and phenotypic differences in different CSCs [8–10]. Experts attending *The Year 2011 Working Conference on CSCs* have suggested that a more accurate conceptual and practical framework of CSCs is important for their elimination [8]. The mechanisms underlying CSC tumorigenesis remain unclear [11]; there is therefore an urgent need for a new CSC theory to improve the understanding of CSC evolution, biology, identification, and to guide the development of effective therapeutic targets.

Recently, Liu reported the dualistic origin of human tumors, suggesting that tumors could arise from blastomeres generated from fertilized eggs, and stem cells generated from reprogrammed somatic cells [12]. This dualistic origin model attempted to explain the malignant characteristics of tumors, and its author claimed its superiority over other tumor origin models. However, this model ignored the dynamic changes that occur in the tumor histopathologic type. Cancers that undergo such changes include not only those that develop from benign tumors, but also those that convert to another histopathologic type after chemotherapy.

In this review, based on the classical CSC model, we propose a monophyletic origin of cancer. This monophyletic model suggests that CSCs are stem cells that lose control of differentiation. Stem cells, including totipotent stem cells, multipotent stem cells, and unipotent stem cells, accumulate crucial mutations that lead to disordered differentiation. Genetic alterations determine the degree of differentiation and whether a tumor is benign or malignant. Next, the model highlights that the primary cause of cancer progression is driven by CSCs. The division and differentiation of CSCs are dominated by tissue repair signals or inflammatory factors. Poorly differentiated cancer cells cannot repair damaged tissues. Thus, upstream signals continuously promote CSCs division and tumor proliferation. A high degree of tumor aggressiveness may correlate with a high-grade atypia in cancer cells. This theory applies to tumors in different tissues and of different pathological types.

## CSCs Originate from Normal Stem Cells (NSCs)

The development of a fertilized egg into adulthood is a complex process. In the first few rounds of embryonic cell division, 2.4 mutations may occur for every generation of cell division [13]. Subsequently, chemical, radial, and inflammatory environmental factors cause DNA damage and tissue injuries [14, 15]. The risk of DNA damage is inherent during cell division and differentiation. Thus, genetic mutations may occur during tissue repair. Current research suggests that oncogenesis requires 3 to 7 crucial mutations to help cancer cells evade cell cycle checkpoints and apoptosis, and gain other malignant biological behaviors [5, 15, 16]. The mutational landscape indicates that normal tissues can usually carry “driver” mutations in cancer genes for decades, the burden of which increases with age [17]. However, only stem cells can self-renew and differentiate to repair damaged tissues throughout their lifespan. By accumulating plenty of oncogenic mutations, NSCs can transform into CSCs [6, 18]. Studies have shown that stem cells and progenitor cells in normal tissues are susceptible to carcinogenic transformation [19]. Tissues such as the intestinal epithelium, airway epithelium, liver, and pancreas are renowned for the strong regenerative ability of their resident stem cells and have a high incidence of cancer [1, 20, 21]. However, few cancers occur in peripheral nervous and myocardium tissue, which may be attributed to a lack of or weak stemness of resident stem cells in these tissues [21, 22].

The transcriptional profile of cancer cells has many similarities with that of stem cells. The activation of stem cell signals, such as WNT, musashi and NOTCH, strongly contributes to cancer heterogeneity, progression, metastasis and therapy resistance [23–25]. NSCs have been considered the most likely source of CSCs. The earliest evidence for this hypothesis was that only hematopoietic stem cells (HSCs) with specific gene mutations could transform into hematological cancers. These HSCs have been used in targeted therapy for the treatment of hematological cancer patients [26, 27]. On the other hand, cancer initiation in young people or during childhood may occur because of key mutations that were inherited or acquired during the embryonic period. For example, patients with congenital heart disease have genetic variants that may increase the risk of cancer [28].

CSCs have been reported in many solid tumors, including breast cancer, colon cancer, glioblastoma, and pancreatic cancer. They play crucial roles in tumorigenesis, tumor growth, chemoresistance, metastasis, and recurrence [29–31]. In monophyletic model, because CSCs develop from NSCs, there are many similarities between CSCs and NSCs in terms of their cellular characteristics, such as their self-renewal ability and apoptosis inhibition programs [32]. Studies have identified various CSC markers, used in the isolation of CSCs, or for diagnostic, prognostic, and therapeutic purposes [33, 34]. However, there is no single specific marker that can be used to distinguish CSCs from cancer cells. Researchers usually use several markers or a combination of surface and intracellular markers to confirm the identity of CSCs. Here, we summarize the most prominent markers of CSCs, resident stem cells, and human embryonic stem cells (hESCs) in several high-incidence cancers, based on the 2022 cancer statistics (Table 1) [33–78].

**Table 1:** The markers of CSCs, NSCs, and hESC

Tissues	CSCs markers	Resident normal stem cells markers	References	hESC markers (33)
Breast	ALDH1, CD29, CD44, CD133, CD201, EpCAM, PODXL1, SSEA3, SSEA4, TRA-1–60, TRA-1–81, Tspan8	Axin2, CD10, CD1d, CD24, CD29, CD49f, CD49b, CD61, CD90, CD133, CK5, CK8, CK14, CK18, CK19, c-Kit, Lrp5, Lgr5, Lgr6, EpCAM, Procr, sca1, Myh11	(33–37)	ABCG2, CD133, CD90, CD326, CD24, CD49f, CD146, CD10, CD117, CD26, CD29, CD9, CD166, Cripto1, Notch2, PODXL1, SSEA1, SSEA3, SSEA4, TRA-1–60, TRA-1–81
Prostate	ABCG2, ALDH1, CD29, CD44, CD49f, CD133, CD151, CD166, Sca1, TRA-1–60	CD29, CD49f, CD133, Sca1, Trop2, Lgr5	(38–42)	
Lung	ABCG2, ALDH1, CD44, CD56, CD133, CD166, Cripto1, Notch2, Notch3, PODXL1, SSEA1, Sox2	ABCA3, ASCL1, α-SMA, CD49f, CD271, CXCR4, GRP, KRT5, KRT8, KRT17, KRT19, LAMP3, NCAD, PDPN, ROBO, SCGB1A1, SCGB3A2, SFTPC, SMMHC, TP63, TM4SF1	(33, 43–45)	
Colorectal	ALDH1, CD26, CD29, CD44, CD133, CD166, Cripto1, EpCAM, Lgr5	Lgr5	(33, 46–49)	
Melanoma of skin	ABCB5, ALDH1, CD20, CD133, CD271, Nanog, Oct3, Oct4	CD29, CD46, CD49f, CD71, DLL1, DSG3, FRMD4A, LRIG1, MCSP	(50–56)	
Renal	ALDH1, CD24, CD44, CD105, CD133, CXCR4, SSEA1	CD13, CD29, CD44, CD73, CD90, CD105, CD133, CD146, CD224, CK7, c-kit, NR3C2, Pax2, Pax8, SSEA4	(57–62)	
Leukemia	ALDH1, CD9, CD25, CD26, CD32, CD33, CD34, CD44, CD47, CD93, CD96, CD97, CD99, CD103, CD123, CLL1, IL1RAP, PODXL1, TIM3	ABCG2, CD133, CD26, CD34, CD44, CD45RA, CD49f, CD90, c-Kit, PODXL1, Sca1	(33, 63–70)	
Pancreas	ABCG2, ALDH1, CD133, CD9, CD24, CD44, CD49f, c-Met, CXCR1,2, CXCR4, EpCAM, GLRX3, Notch2, Nestin, Notch3, Oct3,4, PODXL1, Tspan8	Unsure markers: Cytokeratins, PDX1, Glb1	(33,71–78)	

It is worth noting that most CSC markers are derived from markers present in hESCs. More than half of the CSC markers in prostate, breast, lung, and colorectal cancers are consistent with hESC markers. Existing drugs targeting CSC markers in solid cancers have shown poor efficacy in clinical trials [6, 19]. Another study reported that there were no statistical differences in the expression of several stemness markers between pancreatic cancer tissues and normal pancreas tissues. The expression of CSC markers is not related to tumor grade or differentiation grade [79]. Many pathways involved in NSC self-renewal or stemness maintenance promote the proliferation and invasion of CSCs [7]. This indicates that CSCs transform from NSCs in various tissues. CSC markers may be heterogeneous in cancers of different tissues. However, the specific markers of most normal adult stem cells in different tissues have yet to be identified [80]. Most NSC markers are derived from hESC markers. Thus, the true identity of CSCs developed from NSCs requires further exploration.

## CSCs Play a Leading Role in Cancer Development and Treatment Resistance

Chronic inflammation leads to the destruction of tissues and the activation of wound-healing signals. Prostaglandin E2 (PGE2) is a well-known signaling molecule whose release is induced by inflammation. PGE2 plays a vital role in stem cell differentiation, angiogenesis, and tissue regeneration in the heart, liver, intestine, kidney, and many other organs [81, 82]. PGE2 has been shown to have potent cancer-promoting effects [83, 84]. Notably, a study verified that PGE2 enhances the stemness of colon and gastric CSCs by activating many signaling pathways and promoting cancer metastasis [84, 85]. Other molecules, such as transforming growth factor beta (TGF-β) and yes-associated protein (YAP), have also been reported to regulate wound healing, cancer proliferation, and stemness maintenance [86–88]. YAP is essential for cancer initiation in many solid tumors and may be a potential therapeutic target [89]. Dysregulated Hippo pathway and YAP/TAZ–TEAD activity are reported to be related to tissue regeneration, wound healing, and CSC maintaining [90, 91]. Therefore, the pathways or molecules activated by inflammation during the wound healing process may trigger CSC proliferation and differentiation.

The original purpose of CSCs was to repair injured tissues. However, CSCs become various immature cell types, that is, cancer cells with high heterogeneity. Moreover, undifferentiated cells cannot restore the function of damaged tissues. Thus, the wound-healing signals promoting the proliferation and self-renewal of CSCs are continuously activated and result in a “wound that never heals” [92]. Alternatively, recruited MSCs differentiate into cancer-associated fibroblasts, which remodel the stroma and microenvironment, result in hypoxia, and accelerate angiogenesis and tumor metastasis [93].

**Fig. 1:**
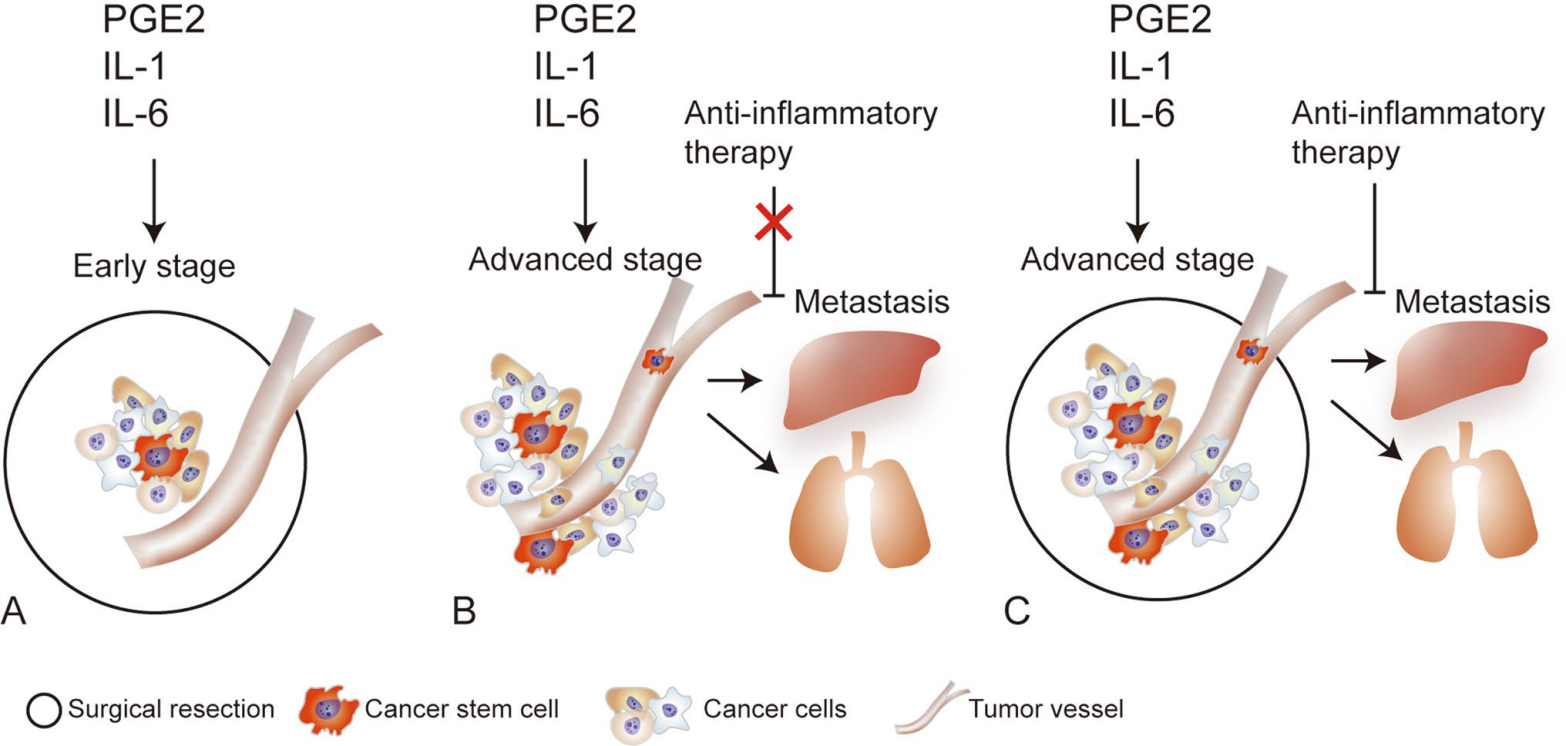
The treatment hypothesis based on monophyletic model. (**A**) Only surgery can completely remove a tumor, including CSCs, in the early stages of cancer. (**B**) Inflammatory factors, such as interleukin (IL)-1, IL-6, and PGE2, play an important role in cancer metastasis. Inflammatory inhibitors cannot prevent metastasis and achieve poor clinical efficacy when the primary tumor is not resected. (**C**) Inhibitors of inflammatory factors play an important role in preventing recurrence and progression after surgical resection

Since CSCs are derived from NSCs, undifferentiated cancer cells are homologous to normal cells to some extent. Even though immature cells are highly heterogeneous, it is difficult for the immune system to detect them. In recent years, next-generation sequencing, multi-omics, and single-cell sequencing technologies have developed rapidly, but very few specific molecular markers for CSCs have been identified [94, 95]. Immunotherapy, including neoantigen-based treatment and chimeric antigen receptor-T cell therapy, has shown limited effectiveness against solid tumors [96, 97]. Chemoresistance is a common characteristic of CSCs [98]. Currently, surgical resection remains an effective treatment for many solid tumors. It is believed that only surgery can completely remove a tumor, including CSCs, in the early stages of cancer (Fig. 1A) [99]. The 5-year relative survival rate associated with many tumors in the localized stage is higher than that associated with tumors in more advanced stages [1]. Many advanced-stage tumors have a poor prognosis, despite R0 resection [100–103]. Under some circumstances, surgical resection may increase the risk of cancer metastasis and progression, owing to systematic inflammation activation, ischemia/reperfusion injury, and immunosuppression [104]. Inflammatory factors, such as TGF-β, interleukin (IL)-1, IL-6, PGE2, and nuclear factor kappa B, have been confirmed to play an important role in cancer metastasis [105–107]. IL-6 has been shown to promote distant metastasis in cancers of the liver, lung, and breast, as well as in many other solid malignancies [108–110]. Tocilizumab, an inhibitor of the IL-6 receptor (IL-6R), suppresses the metastasis and progression of cancer when tested in cell lines and mouse models [111–114]. However, a large clinical randomized trial showed that IL-6R inhibitors are not effective against many cancers [115].

Circulating tumor cells (CTCs) are disseminated into peripheral blood and may lead to distant organ metastasis. Studies have reported that CTCs expressing CSC markers are associated with tumor metastasis and progression [116–118]. We hypothesized that tumors in the advanced stage disseminate CSCs, which initiates cancer metastasis. Inflammatory factors, such as IL-6, might be important accelerators of tumor formation and growth. Since many inflammatory factors are released from primary tumors, IL-6R inhibitors cannot prevent the metastasis induced by IL-6 and achieve poor clinical efficacy when the primary tumor is not resected (Fig. 1B). Occasionally, IL-6R inhibitors may hinder tumor progression. A small source of water is insufficient to extinguish large fires but may prevent reignition; similarly, inhibitors of inflammatory factors may play an important role in preventing recurrence and progression after surgical resection (Fig. 1C).

## CSCs are Essentially Stem Cells with Disordered Differentiation

The concept of CSCs has been established for decades and has been verified by many researchers. If CSCs transform from NSCs and share many critical pathways with NSCs, what are the differences between them? Why do drugs that target CSC markers have poor clinical benefits? In our proposed monophyletic origin model, CSCs are derived from NSCs in different tissues from where the CSCs are located and have almost the same cellular characteristics as NSCs, including surface markers and intercellular pathways. The main biological feature that distinguishes CSCs from NSCs is that CSCs either differentiate into disordered cell types that do not restore the function of damaged tissues or fail to differentiate into mature tissues at all; for example, CSCs in hematopoietic cancers are blocked at a specific differentiation stage [119–121]. According to histopathological classification, the level of differentiation has been used to distinguish benign tumors from malignant ones, characterize the malignant behaviors of cancers, and predict prognosis [122, 123]. Epigenetics has been reported to play a vital role in the regulation of differentiation [124, 125]. Differentiation therapy has proven to be clinically useful for the induction of cancer cell differentiation in leukemia [126]. Therefore, signaling pathways that trigger differentiation failure may be an effective therapeutic target in the termination of CSC activation.

**Fig. 2:**
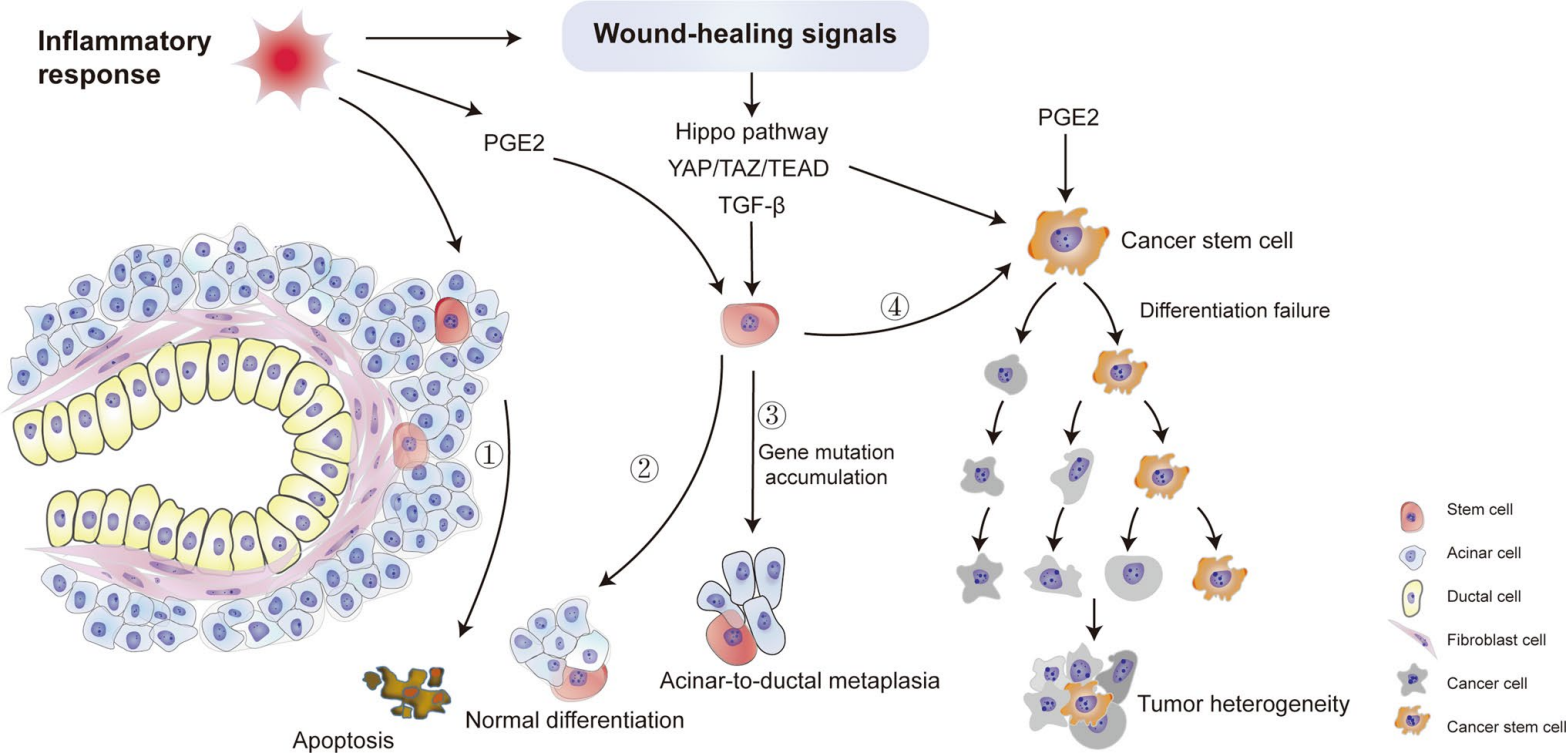
Progression model for pancreatic cancer based on the monophyletic model. Inflammatory responses in the pancreas induce cell apoptosis (①). PGE2 and wound-healing signals are activated to repair the injured tissues through NSC division and differentiation (②). NSCs dedifferentiate and adopt a novel status termed acinar-ductal metaplasia because of the gene mutation accumulation (③). NSCs become malignant CSCs (④). PGE2 and wound-healing signals maintain the division and proliferation of CSCs and lead to tumors

In our proposed model, the development of CSCs from NSCs is a long, continuous process. The most common cancers, such as gastric cancer, which develops from chronic atrophic gastritis; liver cancer induced by viral hepatitis or alcohol; cervical cancer caused by human papilloma virus; and pancreatic cancer resulting from chronic pancreatitis (CP), have been attributed to inflammation and immunoreaction [127–130]. The triggers of cancer are diverse, but chronic inflammation is the most common cause of tumor initiation [14]; pancreatic cancer has the poorest survival of all cancers and has a typical transformation process from CP to oncogenesis. Many studies have reported the key role of CSCs in pancreatic cancer, meanwhile, the pancreas is renowned for its capacity for regeneration [1, 78]. Based on the theory of monophyletic origin, we have proposed that CP progresses to pancreatic cancer; this will provide a better understanding of its progression and is suitable for other tumors (Fig. 2).

In the first stage of CP, alcohol, smoking, and other factors lead to acinar cell injury and inflammatory responses in the pancreas [131]. To repair damaged tissues and restore secretion by acinar cells, quiescent NSCs are activated, start the self-renewal process, and differentiate into acinar cells (Fig. 2). However, DNA damage and gene mutations, such as the Kras mutation that occurs during the repair process, may result in serious DNA damage-repair disorders in NSCs [132, 133]. At this stage, NSCs dedifferentiate and adopt a novel status termed acinar-to-ductal metaplasia (ADM) after the expression of genes regulating acinar lineage–specific transcription factors is inhibited (Fig. 2). However, the secretory functions of acinar cells are maintained in ADM [134, 135]. Metaplasia has also been shown to occur in the esophagus, stomach, lung airway, cervix, and mammary gland, and metaplastic lesions are considered precancerous lesions [132, 136]. Meanwhile, MSCs may be recruited to construct tumor-associated fibroblasts, which promote tumor microenvironment formation and tumor growth [137]. Tumor stromal remodeling induces a hypoxic environment and aggravates tumor progression [138].

If the environmental stress on the pancreas is not alleviated, the tissue repair process progresses to the second stage; NSCs transdifferentiate into a pathological morphology, termed pancreatic intraepithelial neoplasia (PanIN). In this state, some digestive enzyme secretion may be maintained. The degree of dysplasia determines the extent of PanIN lesions, that is, the number of cells with impaired secretory function. Disease progression is halted if the environmental stress is eliminated. However, because of ineffective treatment, many PanIN patients progress to the third stage, in which the NSCs become malignant CSCs (Fig. 2). This example supports the theory that CSCs are stem cells that have lost their ability to differentiate into mature tissues.

## The Relationship Between Monophyletic Origin Theory and the Hallmarks of Cancer

Researchers have identified various characteristics of cancer cells over the past few decades; Hanahan et al. summarized six hallmarks of cancer to establish a logical framework for to categorize tumors and provide a systematic understanding of tumor biological features [139]. Although the six hallmarks have been increased to eight and combined with additional characteristics, the central idea implicit in their theory should be noted: as normal cells develop into a malignant state, they acquire hallmark capabilities [3, 140]. According to the monophyletic origin theory, CSCs that initiate different cancers are transformed from resident NSCs. Thus, rather than acquiring the hallmark capabilities after malignant transformation, CSCs exhibited them before transformed from NSCs. Moreover, the hallmarks of cancer can be easily explained by the theory of monophyletic origin. The eight hallmarks of cancer include sustaining proliferative signaling, evading growth suppressors, resisting cell death, enabling replicative immortality, inducing vasculature, activating invasion and metastasis, reprogramming cellular metabolism, and avoiding immune destruction [140]. According to the monophyletic theory, the division and differentiation of CSCs is a mechanism for repairing injured tissues. Proliferative signaling is sustained by tissue-repair signals or inflammatory factors, because the cells produced by CSCs cannot restore the functions of damaged tissues, or because it is not possible to eliminate inflammatory factors, respectively. Hanahan hypothesized that inflammatory stroma may contribute to the generation and maintenance of CSCs [140]. The hypoxic environment induced by MSCs recruited to the tumor stroma facilitates the metastasis of cancer cells. Inflammatory cells have also been reported to promote cancer cell invasion by activating epithelial-mesenchymal transition [141].

Another hallmark of cancer is immune escape. Most studies have shown that the activity of immune cells in the tumor microenvironment is inhibited. However, immune cells and inflammatory responses may also promote tumorigenesis, metastasis, and progression [14, 142, 143]. Rather than killing tumor cells, immune cells present in the tumor microenvironment may be a manifestation of the inflammatory response [144]. These results indicate that inflammatory responses are the key triggers of cancer hallmarks.

In addition, mounting evidence suggests that the mechanisms orchestrating normal embryogenesis are strikingly similar to those associated with CSCs [145]. For example, telomerase activity in CSCs is consistent with that in NSCs and plays a crucial role in sustaining cell division and proliferation in cancer. Telomerase is considered a potent target for cancer therapy [146]. CSCs can switch between the quiescent and active states to escape chemotherapy [147]. Other features of CSCs, such as self-renewal, anti-apoptosis, and replicative immortality, were also confirmed to be exhibited by NSCs.

The most recently reported hallmarks of cancer indicate that cancer cells exhibit phenotypic plasticity. Cancer cells exhibit a complex, heterogeneous, and transformable pathogenesis that involves dedifferentiation, blocked differentiation, and transdifferentiation [3]. The monophyletic theory proposes that the cancer phenotype is determined by the degree of differentiation deficiency in CSCs. The failure to differentiate leads to diverse immature cancer cell phenotypes. Accumulating evidence suggests that many solid tumors are hierarchically organized. This may be because a single tumor evolves from distinct subpopulations of CSCs [148, 149]. Single-cell sequencing technologies have greatly contributed to our understanding of the origin and diversity of cancer [150].

## The Superiority of the Monophyletic Model in Comparison with the Classical CSC Model

The classical CSC model has been established for decades based on the discovery that only a fraction of the population of cancer cells has the ability to self-renew, extensively proliferate, and form tumors, in both *in vivo* and *in vitro* experiments [151, 152]. The transformation of NSCs induced by genetic and epigenetic alterations may be the primary source of CSCs [7, 153–155]. CSCs have considerable biological similarities with NSCs and play a critical role in the initiation, metastasis, propagation, and therapy resistance of cancer [19]. The classical CSCs model relies on hypothetical origins of CSCs including NSCs, dedifferentiated mature cells, and induced pluripotent cancer cells [156, 157]. There are various controversies surrounding the CSCs model among cancer researchers; however, our monophyletic model scientifically summarizes the aberrant differentiation of cancer cells lead by CSCs, the dynamic evolution of CSCs from NSCs, and the driving role of wound-healing signals in cancer progression.

The monophyletic origin model considers that CSCs which originate from NSCs at different differentiation stages and from different resident tissues, have different abilities regarding multi-lineage differentiation. The degree of differentiation and the cellular activity of stem cells determines the biological aggressiveness and the intra-tumor heterogeneity of CSCs. For example, a mature teratoma can produce a variety of cell types similar to an organoid system, due to the potent differentiation capacity of embryonic stem cells. On the other hand, an immature teratoma dynamically transformed from a mature teratoma has very poor differentiation, and highly malignant biology [158, 159]. The monophyletic model presents the dynamic evolution process from normal tissue, to pre-malignant lesions and finally, malignant cancer. Evidence shows that, as the stemness, proliferative ability, and number of stem cells diminishes with age, the regeneration capacity of tissues reduces, such as of the skin and intestines [160, 161]. Tsang et al. found that younger breast cancer patients have a higher expression of the stem cell marker ALDH1 [162]. Studies indicated that cancers in young people have a more aggressive biological features, and lead to a higher mortality and poorer prognosis compared with cancers in older people [162–165]. These may provide a better understanding of the stemness of NSCs that CSCs transformed from decides the malignancy of the tumor.

The dynamic evolution in the monophyletic model is determined by the retained differentiative capacity of CSCs. The purpose of CSC division and proliferation is to repair damaged tissue; however, gene mutations bring about aberrant differentiation or de-differentiation, and lead to ineffective tissue repair. In cancerous tissue, CSCs may obtain mutations inducing transformation between CSCs and non-CSCs, thus resulting in a complex heterogeneous tumor. Furthermore, the monophyletic origin model proposes triggers of cancer progression including inflammatory response and wound-healing signals, such as PGE2, YAP, NOTCH, and WNT signals, which are necessary signals in normal tissue repair [161]. These signals also play critical roles in oncogenesis and cancer progression by controlling CSC activity. Strong correlations between the mechanisms of cancer growth and wound repair have been proven [166]. The resident stem cells performing wound repair have very similar transcriptomes to CSCs [167]. The monophyletic origin model provides a more comprehensive understanding of CSCs compared to the classical CSC model.

## Potential Therapeutic Targets Based on the Monophyletic Model

Based on the classical CSCs model, researchers mainly focused on the discovery of biomarkers for the identification of and targeted therapies against CSCs. However, no specific biomarkers could be used to screen CSCs accurately until now. The drugs targeting CSCs have shown unsatisfactory results [19]. The monophyletic model proposes that aberrant differentiation is the root cause of cancer. Clarifying the molecular mechanisms of cell differentiation is crucial in facilitating novel cancer therapies [168]. Differentiation therapy has been proven to be an effective treatment method against some types of cancer [88, 169]. In addition, according to the monophyletic model, anti-inflammatory response signals and anti-wound-healing signals are pivotal to treatment success and recurrence prevention. Many biomarkers of CSCs play an important role in the inflammatory response. The signaling pathways regulating the inflammatory response and wound repair are primary factors of CSC activity and cancer progression [104–107]. However, it is important to explore and define the appropriate stage for intervention with anti-inflammatory and anti-wound repair therapy, such as post operation.

## Conclusion

Cancer remains a global threat to human health. Currently, no drugs or therapeutic methods can cure cancer. However, significant progress has been made in the development of targeted therapies, immunotherapy, biological therapy, chemoradiotherapy, and surgical treatment [116, 170, 171]. CSCs are considered tumor-initiating cells because they exhibit self-renewal, treatment resistance, metastasis, and tumor formation capabilities [11]. The monophyletic origin model of cancer provides a concise account of how NSCs can transform into CSCs and how certain trigger factors can promote and sustain proliferation, invasion, and chemoresistance. In our view, the biological behaviors of cancer cells should be described as dynamic, chaotic, and self-preserving, rather than aggressive; cancer cells do not intend to destroy organs. In the past few decades, eliminating cancer cells and CSCs has been the main therapeutic principle behind anti-cancer strategies. It may be time to view the biological features of cancer in a different light and pay more attention to the redifferentiation of CSCs and the factors driving CSC differentiation.

## Data Availability

Not available.
